# A highly contiguous reference genome for *Scalesia gordilloi* (Asteraceae), a critically endangered plant endemic to the Galapagos Islands

**DOI:** 10.46471/gigabyte.192

**Published:** 2026-09-25

**Authors:** Gabriela Pozo, Gonzalo Rivas-Torres, Emilio Vélez-Darquea, Doménica Barragán-Orbe, Maria de Lourdes Torres

**Affiliations:** ^1^ Laboratorio de Biotecnología Vegetal, Colegio de Ciencias Biológicas y Ambientales, Universidad San Francisco de Quito USFQ, Quito, Ecuador; ^2^ Galapagos Science Center, Universidad San Francisco de Quito (USFQ) and University of North Carolina at Chapel Hill, San Cristóbal, Galápagos, Ecuador; ^3^ Colegio de Ciencias Biológicas y Ambientales, Estación de Biodiversidad Tiputini, Universidad San Francisco de Quito USFQ, Quito, Ecuador

## Abstract

*Scalesia gordilloi* is a critically endangered species endemic to San Cristóbal Island in the Galapagos archipelago and represents one of the most unique and vulnerable lineages within the adaptive radiation of the genus *Scalesia*. Despite its evolutionary distinctiveness and conservation importance, no genomic resources have been available for this species. Here, we present the first high-quality reference genome of *S. gordilloi*, generated using Oxford Nanopore long-read sequencing. Across three PromethION R10.4.1 flow cells, we obtained 80.5 Gb of long reads (~22× coverage), which enabled a highly contiguous 3.61 Gb assembly composed of only 549 contigs and an N50 of 106.6 Mb. BUSCO completeness reached 98.6%, with assembly metrics comparable to other high-quality Asteraceae genomes. Repeat annotation revealed that 76.2% of the genome is composed of interspersed elements, dominated by LTR retrotransposons. Structural annotation resulted in 47,913 high-confidence protein-coding genes, consistent with expectations for large, repetitive Asteraceae genomes. This genome provides a critical foundation for conservation genomics, enabling assessments of genetic diversity, inbreeding, and adaptive potential in the species. It further establishes a framework for comparative genomics across the *Scalesia* radiation and supports future efforts to protect and restore one of the most threatened plant lineages of the Galapagos Islands.

## Background

**Figure 1.**
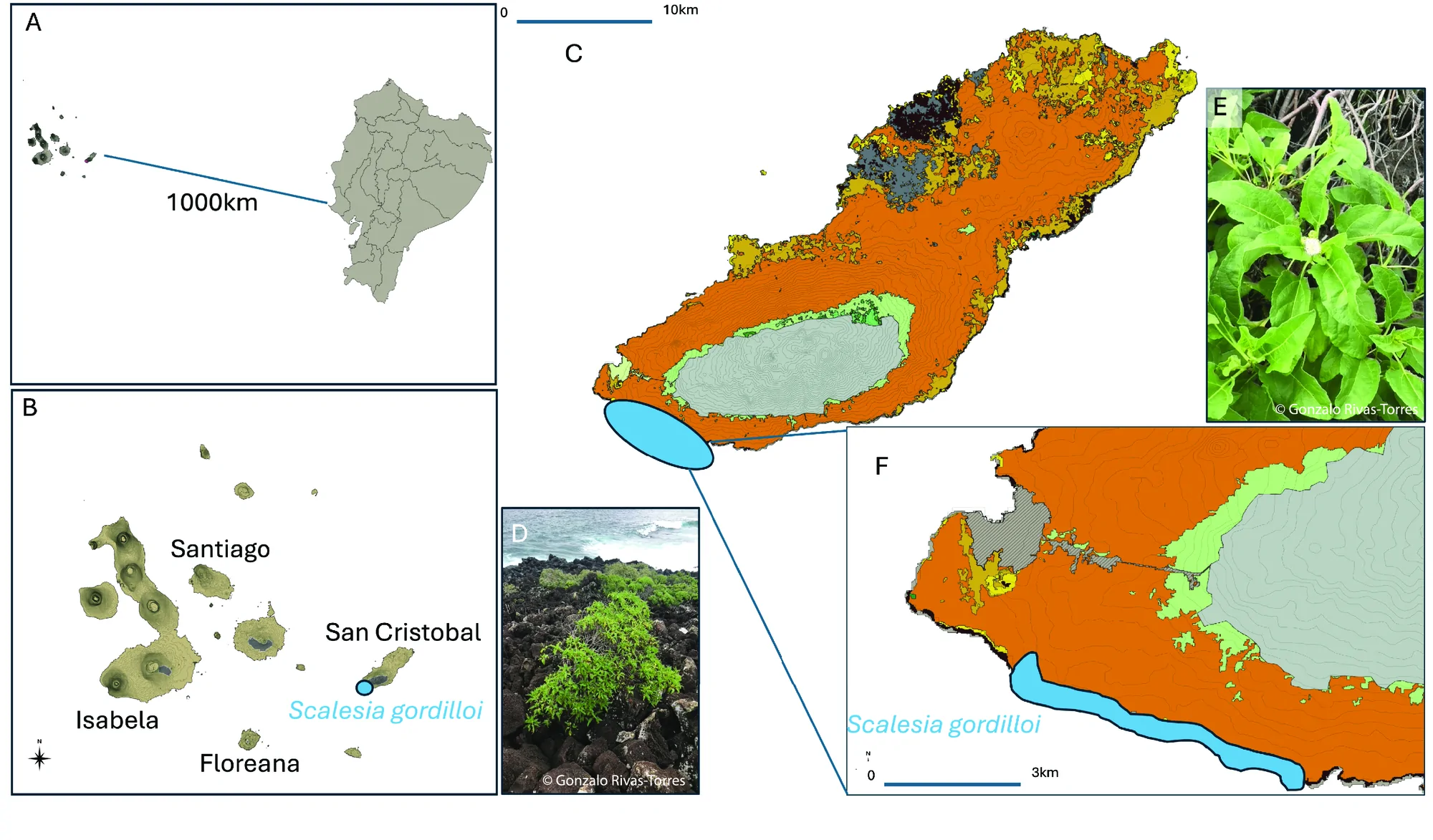
Geographic distribution and habitat of *Scalesia gordilloi* on San Cristóbal Island. (A) Location of the Galapagos Archipelago in relation to mainland Ecuador. (B) Location of San Cristóbal Island in the eastern region of the Galapagos, with Santiago, Isabela, and Floreana Islands shown for reference. (C) Detailed map of San Cristóbal indicating the extent of the Galapagos Dry Forest (in orange) and the approximate distribution of *S. gordilloi* (blue circle). (D) Photograph of an individual of *S. gordilloi* and its native habitat. (E) Close-up image of *S. gordilloi* showing leaf morphology and inflorescence. (F) Close-up map of the known distribution of *S. gordilloi* on San Cristóbal Island.

The *Scalesia *genus (Family Asteraceae, Order Asterales) is an endemic group from the Galapagos archipelago [1]. It is composed of 12 shrubby species and 3 arboreal species that are distributed allopatrically [﻿^1^]. Like Darwin’s finches, species of the genus *Scalesia* are apparently the result of an accelerated process of diversification and adaptation, known as adaptive radiation [2]. This process is common in oceanic islands and has been an important system to study evolutionary mechanisms, since many islands are geologically new [2]. Due to their allopatric distribution, species grow in populations separated from each other, and some are even limited to specific islands, such as *Scalesia gordilloi *[1], which is confined to the southeastern part of San Cristóbal Island (Figure 1).

*Scalesia gordilloi* (Asteraceae/Compositae) was formally described in 1986 [3]. It is characterized as a single-stemmed shrub reaching approximately 1.2–1.5 m maximum in height, although individuals may occasionally exhibit a more prostrate growth form. Its leaves are entire, with petioles measuring approximately 2–3 cm and leaf blades approximately 5–7 cm [3]. The leaves bear glandular structures and, when crushed, release a somewhat pleasant aroma, a characteristic maintained in most of the *Scalesia* species [4].

The species possesses capitulate inflorescences, with individuals being partially heterogamous. The florets are white, and the heads measure approximately 8–10 mm in length (Figure 1D) [3]. The achenes are about 3 mm in maximum length and 1 mm at their widest point, and dispersal occurs primarily by wind.

As mentioned,* S. gordilloi* is a species with a highly restricted distribution, as it has been recorded exclusively on San Cristóbal Island, making it endemic to this island. Within San Cristóbal, *S. gordilloi* occurs in just over 400 hectares in the southwestern portion of the island (G. Rivas-Torres, unpublished data), one of the easternmost and geologically oldest of the Galapagos archipelago (Figure 1C). The species occurs as scattered individuals (approximately every 20–40 m apart) within this range, and is primarily distributed within the Galapagos Deciduous Forest, a dry ecosystem that occupies the lower-elevation perimeter of the islands and is characterized by comparatively lower precipitation, lower relative humidity, and reduced organic soil content than the more humid highland ecosystems [5].

Although *S. gordilloi* is one of four *Scalesia* species present on San Cristóbal, its distribution does not appear to overlap with that of any other *Scalesia* species. *S. gordilloi* is restricted to the southern coast of San Cristóbal, where it occurs in low-elevation arid coastal habitats [3]. In these coastal habitats, it is regularly exposed to sea spray from breaking waves, which often results in the presence of salt crystals on the leaf surfaces. Current population estimates suggest that the species consists of only a few hundred individuals, primarily mature adults (G Rivas-Torres, personal observation). The species is currently listed as Critically Endangered on the IUCN Red List [6]. *S. gordilloi* represents one of the more late-diverging lineages within the genus and is closely related to the more evolutionarily young *Scalesia* species, including *S. divisa* and *S. incisa*, which also occur on San Cristóbal but are found further to the north on the island [2].

The Galapagos Islands harbor a unique biodiversity, being the habitat of iconic species, such as giant tortoises or Darwin's finches [7]. However, the introduction of non-native species represents one of the greatest threats to local populations [8]. In the case of the *Scalesia *genus, which represents one of the most emblematic examples of adaptive radiation in plants in the archipelago, several species are currently threatened or critically endangered, including *S. gordilloi*. Despite their ecological and evolutionary importance, genomic resources for this group remain scarce. To date, *S. atractyloides, *one of the basal lineages of the group*,* appears to be the only species with a high-quality reference genome available [9], highlighting an important gap in genomic knowledge for the genus.

To help address these limitations, several initiatives have emerged aiming to expand genomic resources for biodiversity, particularly in understudied regions. One such effort is ORG.one, which focuses on generating de novo genome assemblies for endangered and critically endangered species using Oxford Nanopore sequencing technologies. These initiatives facilitate the rapid production of genomic data and promote open access to these resources, enabling their use in conservation, evolutionary, and ecological studies [10, 11].

Obtaining a high-quality reference genome is essential for enabling downstream analyses, including the identification of genetic variation, comparative genomics, and phylogenetic inference [12]. Such resources are particularly valuable for species with small and fragmented populations, as they provide critical insights into genetic diversity, population structure, and adaptive potential, all of which are key components for informed conservation strategies.

To address this gap, we generated a comprehensive genomic dataset for *S. gordilloi* using Oxford Nanopore long-read sequencing as part of the ORG.one initiative. This study provides not only a high-quality reference genome assembly, but also associated raw sequencing data and genome annotation resources. A detailed description of these datasets, including their generation, quality, and potential applications, is provided in the following section.

## Data description

This study generated a comprehensive genomic dataset for *Scalesia gordilloi*, including raw long-read sequencing data, a de novo genome assembly, and a structural and functional genome annotation. The assembled genome spans 3.61 Gb and is composed of 549 contigs, with a contig N50 of 106.6 Mb, indicating a highly contiguous assembly. Genome completeness was assessed using BUSCO, recovering 98.6% of conserved single-copy orthologs. Repetitive element annotation revealed that approximately 76.2% of the genome consists of interspersed repeats, primarily long terminal repeat (LTR) retrotransposons.

Structural annotation resulted in 47,913 high-confidence protein-coding genes following filtering steps to remove short and transposable element–associated predictions. The resulting gene models include exon–intron structures consistent with other large Asteraceae genomes.

These datasets provide a valuable resource for multiple downstream applications, including comparative genomics, studies of genome evolution within the Asteraceae, and conservation genomics of endangered island species. In particular, the availability of a high-quality reference genome enables future analyses of genetic diversity, population structure, and adaptive variation in *S. gordilloi *and related taxa.

All datasets associated with this study, including raw sequencing reads, genome assembly, and structural and functional annotation files, are available through public repositories (see Data Availability section), ensuring accessibility and reuse by the research community.

## Analyses

### Sequencing output and data quality

To assess sequencing output and dataset quality, we generated long-read data using R10.4.1 flow cells on the Oxford Nanopore PromethION 2 Solo platform, obtaining a total of 80.5 Gb of raw sequence data across 8.4 million reads. The dataset showed a mean read length of 9.6 kb (median 7.2 kb), a read length N50 of 15.0 kb, and a mean read quality of 12.9 (median 15.2). Based on the final assembly size of 3.61 Gb, the dataset represents approximately 22× long-read coverage.

### Genome assembly and assembly completeness

**Figure 2.**
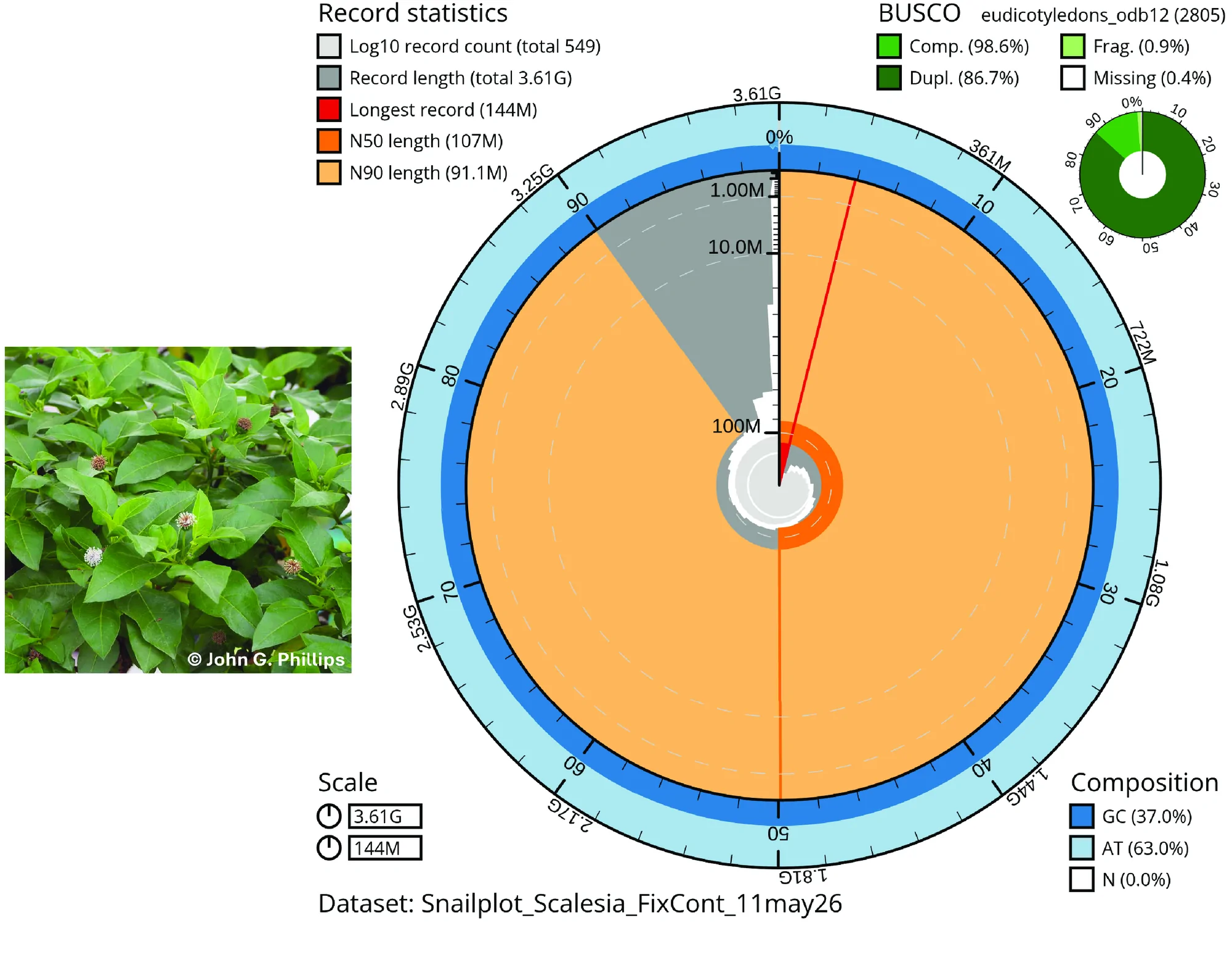
Snailplot summary statistics of the *Scalesia gordilloi* reference genome assembly. Summary shows a total assembly size of 3.61 Gb distributed across 549 contigs. The longest scaffold is 144 Mb, with an N50 of 107 Mb and N90 of 91.1 Mb, indicating a highly contiguous assembly. BUSCO analysis using the eudicotyledons dataset recovered 98.6% complete genes (86.7% duplicated), with 0.9% fragmented and 0.4% missing. GC content is 37.0% and AT content is 63.0%.

**Figure 3.**
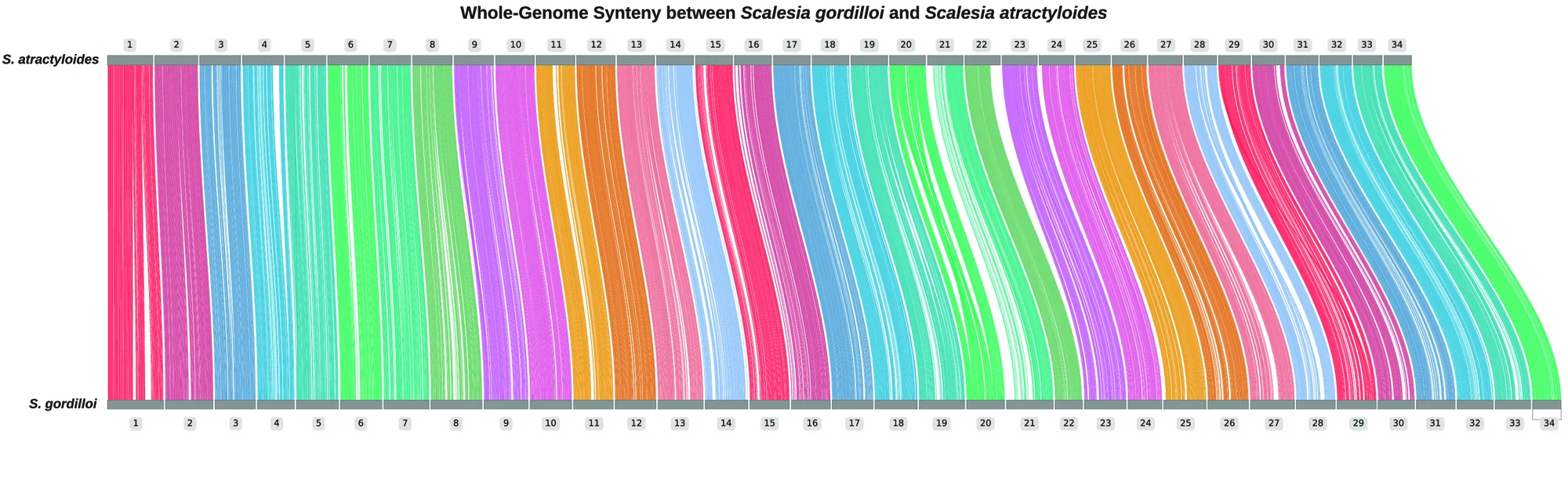
Chromosome-scale genome synteny between *Scalesia gordilloi* and *Scalesia atractyloides*. The 34 pseudochromosomes of *S. atractyloides* (top) and *S. gordilloi* (bottom) are shown in gray. Colored ribbons represent aligned genomic regions between corresponding pseudochromosomes, with colors distinguishing chromosome pairs. Whole-genome alignments were summarized in non-overlapping 1-Mb windows along the *S. atractyloides* reference genome, and windows with ≥50% aligned sequence were retained for visualization. Ribbon orientation represents the predominant alignment orientation within each window.

To generate a high-quality reference genome for *Scalesia gordilloi*, we performed de novo assembly using Hifiasm, which yielded a total genome size of 3.61 Gb, distributed across 549 contigs (Table 1, Figure 2). The longest contig measured 143.7 Mb, with an N50 of 106.6 Mb and L50 of 16, indicating a highly contiguous assembly. Whole-genome comparison with the chromosome-level *S. atractyloides* reference (GCA_947069175.1) showed that 82.2% of the *S. gordilloi* assembly aligned to the reference genome. Chromosome-scale synteny analysis further revealed extensive correspondence across all 34 pseudochromosome pairs, indicating broad conservation of large-scale genome organization between the two species (Figure 3). Unaligned regions in both genomes were predominantly composed of repetitive sequences (>83%), suggesting that repetitive DNA accounts for a substantial proportion of the genomic sequence not captured by the whole-genome alignment (Table 2).

**Table 1:** Genome assembly and BUSCO statistics for *Scalesia gordilloi*.

Statistic	Assembly
Contig number	549
Longest contig	143.7 Mb
Total Length	3.61 Gb
N50	106.6 Mb
L50	16
Genome fraction*	82.2%
Complete BUSCOs**	98.6%
Single-copy	12%
Duplicated	86.7%
Fragmented BUSCOs	0.9%
Missing BUSCOs	0.4%

*Percentage of genome that aligns to reference Scalesia atractyloides. **Database eudicots_odb10 with 2805 orthologous genes.

**Table 2:** Comparative genomic features and whole-genome alignment between *Scalesia gordilloi* and *Scalesia atractyloides*.

Metric	*S. gordilloi*	*S. atractyloides*
Assembly size	3.61 Gb	3.22 Gb
Sequence represented in 34 pseudochromosomes	3.57 Gb (98.78%)	3.18 Gb (98.96%)
Repetitive sequences	76.21%	76.22%
Genome fraction aligned to *S. atractyloides*	82.2%	—
Repeats within unaligned regions	83.68%	84.74%

To evaluate assembly completeness, BUSCO analysis recovered 98.6%, with only 0.4% of genes missing, consistent with a near-complete representation of the *S. gordilloi* genome (Table 1, Figure 2).

Compared with other published Asteraceae genomes, the *S. gordilloi* assembly showed high contiguity and completeness (Table 3). Its N50 of 106.6 Mb was comparable to that of the chromosome-level* S. atractyloides* assembly (94.2 Mb), while BUSCO completeness (98.6%) was similar to or higher than that reported for *S. atractyloides*, *Helianthus annuus*, and *Lactuca sativa* var. *crispa* [9, 13, 14]. These comparisons support the high completeness and contiguity of the *S. gordilloi* assembly despite the absence of chromosome-level scaffolding.

**Table 3:** Genome Assembly and BUSCO statistics for other Asteraceae.

Statistic	*S. gordilloi*	Assembly (*S. atractyloides*)	Assembly *(Helianthus annus *OXS*)*	Asembly (*Lactuca sativa* var. crispa)
Contig number	549	34 (Chromosome models)	107 contigs 82 scaffolds	77 Scaffolds
Longest contig	143.7 Mb	116.23 Mb (Largest Scaffold)	Not reported	407.16 Mb (Largest Scaffold)
Total length	3.61 Gb	3.22 Gb	3.03 Gb	2.59 Gb
N50	106.6 Mb	94.2 Mb	178.40 Mb	320.76 Mb (Scaffold N50)
L50	16	16	8	Not reported
Complete BUSCOs	98.6%	93.3%	98.2%	98.39%
Single-copy	12%	36.3%	85.2%	96.10%
Duplicated	86.7%	57%	13%	2.29%
Fragmented BUSCOs	0.9%	2.8%	0.6%	0.5%
Missing BUSCOs	0.4%	3.9%	1.2%	1.12%

### Repeat annotation

To characterize genome composition, we identified repetitive elements, which accounted for 76.2% of the *S. gordilloi* genome (Table 4). Among these, retroelements represented the dominant class, representing 39.6% of the genome, followed by unclassified repeats (34.9%) and DNA transposons (1.7%). Long terminal repeat (LTR) elements were particularly abundant, especially Gypsy/DIRS1 and Ty1/Copia families, comprising 23.8% and 11.0%, respectively.

**Table 4:** Repetitive elements in the *Scalesia gordilloi* reference genome.

Element	Number of elements	Percentage of sequence
Retroelements	978,057	39.61%
SINEs	92,977	1.7%
LINEs	41,626	0.75%
LTR elements Ty1/Copia Gypsy/DIRS1	843,454 255,095 515,457	37.18% 11.02% 23.80%
DNA transposons	123,810	1.7%
Rolling-circles	9,576	0.12%
Unclassified	3,617,436	34.91%
TOTAL interspersed repeats		76.21%

### Structural annotation

To characterize gene content, structural annotation of the filtered genome yielded 47,913 protein-coding genes, each represented by a single transcript. Genes contained an average of 5.4 exons and 4.4 introns, with mean exon and intron lengths of 308 bp and 332 bp, respectively (Table 5). Coding sequences averaged 1674 bp, and gene models ranged from 906 bp to 59.9 kb in total length. A total of 9342 genes (19.5%) were single-exon models. The final annotation encompassed 80.2 Mb of coding sequences, representing the coding fraction of the assembled genome.

**Table 5:** Structural genome annotation statistics.

Statistic	Value
Number of genes	47,913
Number of exons	259,933
Number of introns in CDS	212,020
Mean exons per gene	5.4
Mean introns per gene	4.4
Mean gene length (bp)	3,145 bp
Gene size range	906–59,899 bp
Mean exon length (bp)	308 bp
Mean intron length (bp)	332 bp
Single-exon genes	9,342 (19.5%)
Mean CDS length	1,674 bp
Longest CDS	15,231 bp
Total CDS length	80.2 Mb

### Functional annotation

Functional annotation of the final set of 47,913 predicted proteins identified sequence similarity and functional information for a substantial proportion of the gene models. DIAMOND BLASTP searches against Swiss-Prot produced matches for 38,650 proteins (80.7%), while 38,621 proteins (80.6%) were associated with GO terms during GO Mapping. GO Annotation classification assigned 35,353 proteins (73.8% of the predicted protein set) to broad functional categories.

**Figure 4.**
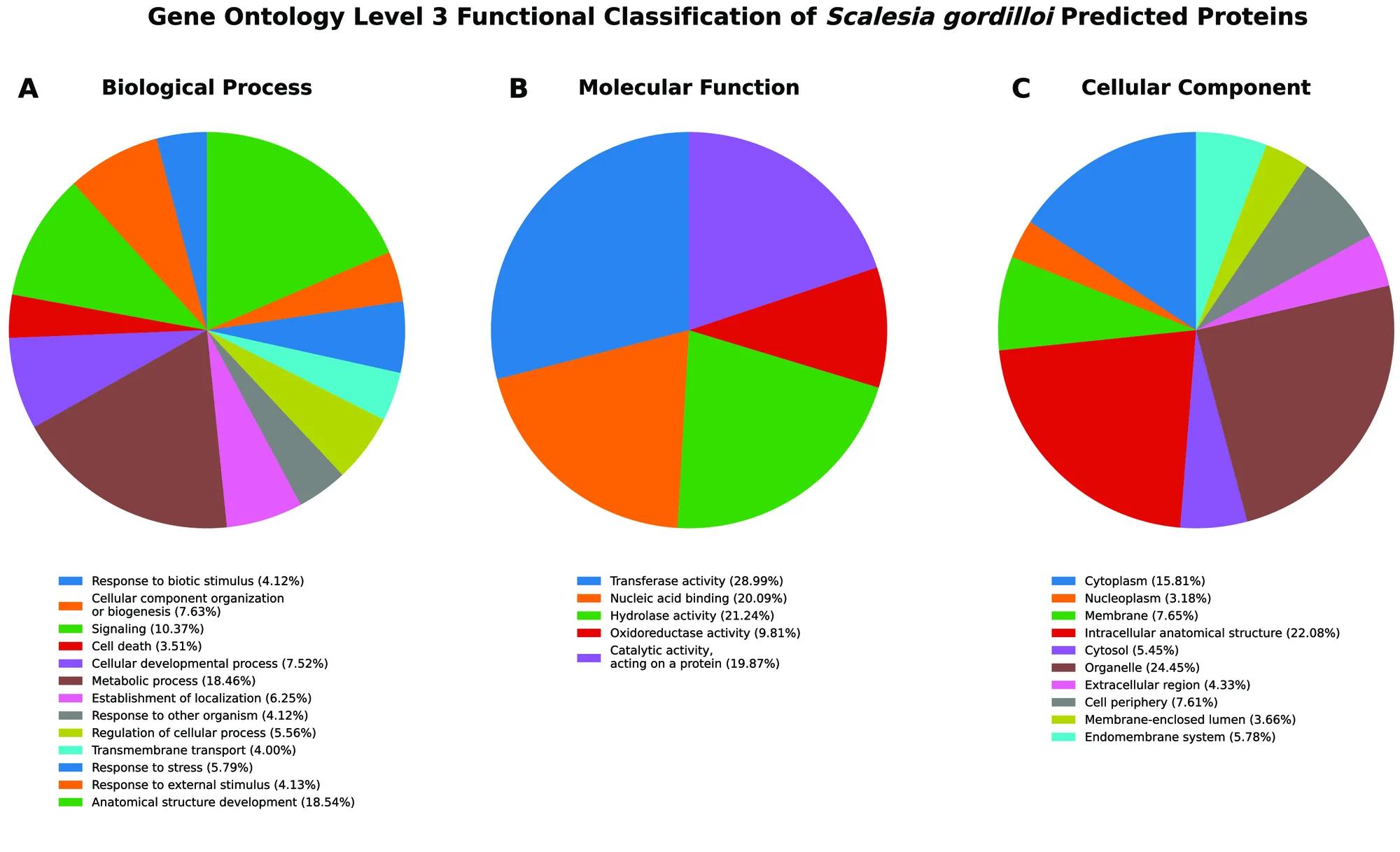
Gene Ontology functional classification of the predicted proteome of *Scalesia gordilloi*. GO Slim level 3 classification of the functionally annotated proteins according to (A) Biological Process, (B) Molecular Function, and (C) Cellular Component. The most represented Biological Process categories included anatomical structure development and metabolic process, while Molecular Function was dominated by transferase activity, hydrolase activity, nucleic acid binding, and catalytic activity acting on a protein. Cellular Component annotations were primarily associated with organelles, intracellular anatomical structures, and the cytoplasm. Percentages represent the relative distribution of sequences assigned to each GO Slim category.

At GO level 3, the most represented Biological Process categories included anatomical structure development (17,038 sequences), metabolic process (16,957), signaling (9524), cellular component organization or biogenesis (7008), and cellular developmental process (6912) (Figure 4). For Molecular Function, the predominant categories were transferase activity (9943), hydrolase activity (7285), nucleic acid binding (6891), catalytic activity acting on a protein (6815), and oxidoreductase activity (3363) (Figure 4). Cellular Component annotations were dominated by organelle (28,949), intracellular anatomical structure (26,144), cytoplasm (18,715), membrane (9056), and cell periphery (9005) categories (Figure 4).

## Discussion

Oxford Nanopore sequencing produced 80.5 Gb of long-read data across 8.4 million reads, equivalent to ~22× coverage of the estimated *Scalesia gordilloi* genome. The dataset showed a mean read length of 9.6 kb, a read N50 of 15 kb, and a mean quality score of Q12.9, values consistent with high-yield PromethION R10.4.1 runs and suitable for assembling large, repeat-rich plant genomes [15, 16]. Long reads are particularly advantageous for spanning repetitive regions, which dominate Asteraceae genomes and which was later shown to constitute ~76% of the *S. gordilloi* genome. Overall, these sequencing metrics provided sufficient depth and read quality for generating a high-contiguity de novo assembly.

The final *S. gordilloi* assembly is highly contiguous, with a total size of 3.61 Gb, only 549 contigs, and an N50 of 106.6 Mb. These metrics represent a substantial improvement over earlier drafts and approach the contiguity of the chromosome-level genome of *S. atractyloides* [9]. BUSCO completeness was 98.6%, with only 0.4% missing genes, matching the completeness of other high-quality Asteraceae genomes such as sunflower and lettuce [15, 16]. The high proportion of duplicated BUSCOs may partly reflect the complex history of whole-genome duplication and genome expansion reported across the Asteraceae, including *Scalesia* and other members of the family [9, 15–18], although residual allelic duplication or assembly redundancy cannot be completely excluded. Together, these metrics indicate that the assembly provides a highly complete and contiguous representation of the *S. gordilloi* genome. Although two rounds of long-read consensus polishing were performed with Racon using the available long reads, no independent short-read dataset was available for additional polishing. Therefore, residual base-level errors characteristic of long-read-only assemblies cannot be completely excluded and may affect a subset of predicted coding sequences. Future incorporation of independent high-accuracy sequencing data could further refine base-level consensus accuracy.

Whole-genome comparison with *S. atractyloides* provides additional evidence of broad genomic conservation within the genus. Approximately 82.2% of the *S. gordilloi* assembly aligned to the *S. atractyloides* reference, while chromosome-scale synteny analysis revealed extensive correspondence across all 34 pseudochromosome pairs. Together, the high proportion of the assembly represented in these pseudochromosomes (98.78%), the extensive syntenic correspondence, and the high BUSCO completeness support the overall continuity and completeness of the *S. gordilloi* genome assembly. However, because the *S. atractyloides* genome was also used as the reference for RagTag scaffolding, the observed chromosome-scale correspondence should not be considered fully independent validation of scaffold structure. Instead, it demonstrates that the resulting assembly is broadly consistent with the chromosome-scale organization of the available congeneric reference. The final *S. gordilloi* annotation comprises 47,913 protein-coding genes, a number consistent with expectations for large, highly repetitive genomes within the Asteraceae, many of which contain between 40,000 and 55,000 genes due to extensive transposable-element expansion and historical whole-genome duplications [15, 16]. This gene count also closely matches that of *S. atractyloides*, the only previously published chromosome-level genome for the genus. In that species, Cerca et al. [9] reported 46,375 Iso-Seq–supported genes and 43,093 final annotated, providing a strong benchmark for the genomic architecture of *Scalesia *[9]. Overall, the similarity in gene number between the two *Scalesia* species is consistent with broadly comparable genome organization, although additional genus-wide analyses would be necessary to evaluate whether this reflects shared evolutionary patterns across the genus.

The annotation statistics of *S. gordilloi* are fully consistent with other Asteraceae genomes, which are characterized by high repeat content (>70%) and large gene repertoires. For example, sunflower (*Helianthus annuus*) contains ~52,000 genes and 74% repeats [15], lettuce (*Lactuca sativa*) contains ~39,000 genes and 74% repeats [16], and the Hawaiian *Bidens hawaiensis *genome harbors 74% repetitive DNA [17]. The ~76% repeat content and ~48k genes recovered in *S. gordilloi* therefore fall well within the expected variation for this family.

The structural characteristics of the predicted gene set are broadly consistent with those reported for other Asteraceae genomes [15, 16, 18]. However, an important limitation of the current annotation is the absence of species-specific transcriptomic evidence. Although protein evidence from Heliantheae species was incorporated and post-processing filters were applied to remove short and transposable element-associated predictions, RNA-seq or Iso-Seq data from *S. gordilloi* were not available to directly validate transcript structures. Consequently, some exon–intron boundaries, splice sites, and individual gene models may require further refinement. Future incorporation of species-specific transcriptomic data would enable validation of gene structures, identification of alternative isoforms, and further improvement of annotation accuracy.

Functional annotation further supports the utility of the predicted gene set, with approximately 81% of the predicted proteins producing significant matches against the curated Swiss-Prot database and 73.8% receiving GO Slim classifications. The representation of genes associated with metabolic processes, development, signaling, catalytic activities, and major cellular compartments indicates broad functional coverage of the predicted proteome. These annotations substantially increase the usability of the genome resource by linking the predicted gene models to putative biological functions and provide a foundation for future comparative and conservation genomic analyses in *Scalesia*.

According to this study, *S. gordilloi* and its more distantly related congener *S. atractyloides* share approximately 82.2% of their genome. While direct genome-wide similarity estimates are rarely reported for plants, studies in Asteraceae and other angiosperms indicate that closely related species often exhibit high gene-level similarity (typically 85–95% identity among orthologs) alongside substantial structural variation [15, 16, 19]. Therefore, this value likely reflects a moderate-to-high level of genomic similarity, although differences in methodology and assembly quality limit direct comparisons. However, despite this genomic similarity, the most recent and comprehensive phylogenetic analyses indicate that although they share a common ancestor, these species do not cluster in closely related clades. *S. atractyloides* is more closely related to *S. stewartii*, and together these two species form a monophyletic clade that is evolutionarily closer to the ancestral and more basal species *S. villosa *[2]. Moreover, *S. atractyloides* and *S. gordilloi* occur on islands that are geographically distant from one another. *S. atractyloides* is distributed in the southwestern region of Santiago Island, which lies approximately 152 km northwest of the *S. gordilloi* population on San Cristóbal. This distance is separated almost entirely by open ocean. However, we did not directly assess large-scale genomic structure; additional analyses such as synteny would be needed to evaluate structural conservation across the genus.

Overall, the *S. gordilloi* genome and gene models constitute a robust and well-supported genomic resource, exhibiting characteristics comparable to those reported for other published Asteraceae genomes. This high-quality resource provides a strong foundation for future studies of comparative genomics, adaptive evolution, and island diversification within this endemic Galapagos lineage.

This study also provides a scalable methodology for generating reference genomes of additional *Scalesia* species. A clear next step is sequencing the close relatives of *S. gordilloi*, *S. incisa* and *S. divisa*, which are listed as Vulnerable and Critically Endangered, respectively [20, 21]. Obtaining high-quality genomes for these species would refine their phylogenetic relationships, improve assessments of genomic diversity and conservation value, and enable identification of genes linked to ecological specialization. Such genomic resources will be critical for informing conservation actions, predicting adaptive potential under climate change, and guiding restoration strategies across the genus.

## Potential Implications

The high-quality reference genome of *Scalesia gordilloi* provides a valuable resource that can be applied beyond the scope of this study. In conservation genomics, this dataset enables future analyses of genetic diversity, inbreeding, adaptive variation, and population structure in one of the most threatened plant species of the Galapagos Islands. The increasing availability of genomic resources for endangered plant species further demonstrates the value of reference genomes for evaluating genomic vulnerability, adaptive potential, and conservation priorities [22, 23]. Such information will be essential for informing evidence-based conservation strategies, including habitat restoration, population monitoring, ex situ conservation, and potential assisted gene flow approaches. In addition, the availability of a reference genome may support the identification of genetically vulnerable populations and help guide conservation actions aimed at preserving the evolutionary potential and long-term resilience of the species under ongoing environmental change.

More broadly, this genome contributes to comparative genomic studies within the Asteraceae, a family characterized by large and highly repetitive genomes. The availability of this resource will facilitate investigations into genome evolution, transposable element dynamics, and adaptation in island plant radiations. In particular, comparative analyses across the *Scalesia* genus may help elucidate the genomic basis of adaptive radiation in oceanic island systems.

Finally, this study demonstrates a scalable approach for generating high-quality reference genomes for other endemic and endangered plant species using long-read sequencing technologies. The methodologies and datasets presented here can support future genomic efforts in biodiversity-rich but underrepresented regions, promoting the integration of genomics into conservation and evolutionary research.

## Methods

### Sample collection

Seven adult individuals of *Scalesia gordilloi*  were sampled within the known distribution range of the species. Each sampled plant was located at least 20 m apart to ensure independence of collections. From each individual, 3–5 young leaves were collected and immediately placed in airtight bags with moist paper to maintain tissue hydration. These bags were then placed in cooled containers in the field to maintain low temperature conditions.

The samples were transported to the Galapagos Science Center, where they were stored at –20 °C. Samples were subsequently transported in sealed cold containers to the Plant Biotechnology Laboratories of USFQ in Quito. All sampling, handling, and DNA extraction procedures were conducted under Research Permit No. MAAE-DBI-CM-2021-0198. No individuals were harmed during sampling, as care was taken to avoid damaging branches or trampling other plants within the population.

### DNA extraction, library preparation, and sequencing

High molecular weight DNA was obtained from fresh young leaf tissue of a unique *S. gordilloi *specimen using the Blood & Cell Culture DNA Kit (Qiagen) and a modified CTAB protocol [24] starting with 1 g and 100 mg of tissue, respectively. DNA was eluted in 100 µl, and fragments shorter than 25 kb were removed using the Circulomics Short Read Eliminator Kit (PacBio). Finally, DNA concentration was measured using a Qubit 4 Fluorometer and DNA integrity was evaluated with electrophoresis on a 1.5% agarose gel.

The Ligation Sequencing Kit V14 (SQK-LSK114; Oxford Nanopore Technologies) was used to prepare one sequencing library for each DNA extraction method, using 1000 ng of initial DNA material. The libraries were prepared according to the manufacturer’s protocol for the Ligation Sequencing Kit V14, with a few small modifications, specifically during the DNA repair and end-prep step (15 minutes of incubation at 20 °C, 15 minutes at 65 °C, and a single ethanol wash), and the adapter ligation and clean-up step (20 minutes of incubation at room temperature and a single wash with long fragment buffer).

Sequencing was carried out on a PromethION 2 Solo using R10.4.1 flow cells across three runs lasting approximately 24, 46 and 66 hours. MinKNOW (v6.5.14) was used for data collection, and high-accuracy basecalling was performed by Dorado (v7.9.8) with a minimum Q score of 7.

The published genome size of the congeneric species *S. atractyloides* (3.2 Gb) was initially used as an approximate reference for sequencing-depth planning. An independent genome-size estimate was not generated for *S. gordilloi* because no complementary high-accuracy short-read dataset suitable for conventional k-mer-based genome-size estimation was available.

### Genome assembly and analyses

All reads that passed the Q7 threshold were concatenated prior to adapter removal with Porechop v0.2.3_seqan2.1.1 [25]. Read quality and length were evaluated using NanoPlot v1.32.1, and dataset cleanup was performed with NanoFilt v2.3.0 [26] by removing reads shorter than 1000 bp. The resulting filtered dataset was used for the *de novo* genome assembly of* S. gordilloi *using Hifiasm v0.25.0-r726 [27], executed with ONT-specific settings. Raw reads were subsequently aligned to the draft assembly using minimap2 v2.30-r1287 [28], and two iterative rounds of polishing were performed with Racon v1.4.20 [29]. Finally, duplicated contigs resulting from haplotypes in polyploid genomes were identified and removed with Purge Haplotigs v1.1.3 [30]. The polished and haplotig-purged assembly was subsequently scaffolded with RagTag v2.1.0 using the published *S. atractyloides* genome (GCA_947069175.1) as the reference. Assembly completeness was assessed using BUSCO v5.8.3 [31], and assembly statistics were generated with QUAST v5.0.2 [32].

Comparative genomic analysis between *S. gordilloi* and *S. atractyloides* was performed using MUMmer4, with *S. atractyloides* (GCA_947069175.1) as the reference and *S. gordilloi* as the query. Whole-genome alignments were generated with NUCmer using maximal matches (–maxmatch), a minimum match length of 100 bp (-l 100), and a minimum cluster length of 500 bp (-c 500). For chromosome-scale synteny analysis, the 34 pseudochromosomes of each assembly were paired according to their predominant whole-genome correspondence and renamed consistently from chr1 to chr34. Alignments were filtered using delta-filter -m to retain matches with ≥90% sequence identity and ≥1 kb alignment length. For visualization, alignments between corresponding pseudochromosomes were summarized in non-overlapping 1-Mb windows along the *S. atractyloides* reference genome. Alignment coverage within each window was calculated from the union of aligned intervals to avoid double-counting overlapping matches, and windows with ≥50% aligned sequence were retained. The corresponding position in *S. gordilloi* was determined from the median query position of alignments within each window, and ribbon orientation was assigned according to the predominant alignment orientation weighted by alignment length.

### Genome annotation and repetitive elements analysis

Structural gene annotation was performed using the soft-masked *S. gordilloi* genome produced with RepeatModeler2 (v2.0.7) [33] and RepeatMasker (v4.1.9) [34]. Gene prediction was conducted in OmicsBox v3.4.5 (BioBam Bioinformatics, Valencia, Spain) using the Genome Analysis module with *Arabidopsis thaliana *selected as the closest annotated organism to guide *ab initio *predictions. As external protein evidence, we incorporated a curated set of Heliantheae protein sequences downloaded from NCBI, reflecting the closest available high-quality proteomes within the Asteraceae. These datasets were used by OmicsBox to refine intron–exon boundaries and improve gene model support in the highly repetitive *S. gordilloi* genome. The initial structural annotation was exported as a GFF3 file containing predicted genes, transcripts, exons, and CDS features, and subsequently underwent the post-processing filtering described below to generate the final structural annotation dataset.

An initial InterProScan [35] analysis was performed on the predicted protein sequences to support post-processing of the structural annotation. To improve the biological accuracy of the annotation and reduce inflation arising from fragmented or transposable-element–derived predictions, we applied a two-step filtering procedure using AGAT v1.6.1 [36]. First, we removed all gene models whose total CDS length was <300 bp to eliminate truncated ORFs and spurious short predictions. Second, the initial InterProScan output was parsed to identify proteins associated with transposable element activity (for example transposases, integrases, retrotransposon-related domains) and removed the corresponding transcript and gene models from the GFF3. This filtering yielded a final set of 47,913 protein-coding gene models. The corresponding filtered coordinate, CDS, and protein sequence files were generated by retaining only transcript IDs present in the final filtered GFF3, ensuring consistency among the structural annotation, coding sequences, and predicted proteins. The resulting filtered GFF3, coordinate, CDS, and protein sequence files constitute the final structural annotation dataset.

The final filtered set of 47,913 predicted proteins was subsequently subjected to a complete functional annotation workflow in OmicsBox. Protein similarity searches were performed using DIAMOND BLASTP against the Swiss-Prot database (2026-07-02), and InterProScan was rerun on the filtered protein set to identify protein domains and families and retrieve associated InterPro entries and Gene Ontology (GO) terms. The resulting BLAST and InterProScan evidence were integrated through GO Mapping and GO Annotation, followed by GO Slim analysis to provide a broader functional classification of the annotated proteins. Following this workflow, the functionally annotated sequences and gene models were exported in FASTA and GFF3 formats, respectively, with functional information incorporated into the corresponding sequence and feature annotations. A complete functional annotation table was additionally exported in TSV format. The filtered structural annotation files and the resulting functionally annotated FASTA, GFF3, and TSV files were deposited in *GigaScience* repository.

## Data Availability

The raw sequencing reads generated in this study are available in the NCBI Sequence Read Archive (SRA) under accession number SRR30429356, within BioProject PRJNA1152989. The genome assembly has been deposited in NCBI under BioProject PRJNA1152989. All additional supporting data [37] are available in the *GigaScience* repository, GigaDB [38].

## List of abbreviations

- bp: Base pairs
- BUSCO: Benchmarking Universal Single-Copy Orthologs
- CDS: Coding Sequence
- Gb: Gigabases
- kb: Kilobases
- LTR: Long Terminal Repeat
- Mb: Megabases
- ONT: Oxford Nanopore Technologies
- SRA: Sequence Read Archive
- TE: Transposable Element

## Declarations

### AI use statement

Generative artificial intelligence (AI) tools were used during manuscript preparation to assist with language refinement, grammar correction, and improvement of readability. No AI tools were used for data generation, analysis, interpretation, or drawing scientific conclusions. All outputs generated with AI assistance were carefully reviewed and edited by the authors, who take full responsibility for the accuracy and integrity of the final manuscript.
