# Supplementary material for: A highly contiguous reference genome for Scalesia gordilloi (Asteraceae), a critically endangered plant endemic to the Galapagos Islands
Source: GigaByte. 2026 Sep 25;2026:gigabyte192. doi: 10.46471/gigabyte.192 (PMC13683956; doi:10.46471/gigabyte.192)
Supplement: Peer review history

# Reviewer report: Review MS


Reviewer name and names of any other individual's who aided in reviewer Yunyun LvDo you understand and agree to our policy of having open and named reviews, and having your review included with the published papers. (If no, please inform the editor that you cannot review this manuscript.)YesIs the language of sufficient quality?YesPlease add additional comments on language quality to clarify if needed
Are all data available and do they match the descriptions in the paper? YesAdditional CommentsAre the data and metadata consistent with relevant minimum information or reporting standards? See GigaDB checklists for examples <a href="http://gigadb.org/site/guide" target="_blank">http://gigadb.org/site/guide</a>YesAdditional CommentsIs the data acquisition clear, complete and methodologically sound?YesAdditional CommentsIs there sufficient detail in the methods and data-processing steps to allow reproduction?YesAdditional CommentsIs there sufficient data validation and statistical analyses of data quality? YesAdditional CommentsIs the validation suitable for this type of data?YesAdditional CommentsIs there sufficient information for others to reuse this dataset or integrate it with other data?YesAdditional CommentsAny Additional Overall Comments to the AuthorThis manuscript presents a valuable genomic resource for the critically endangered Scalesia gordilloi. The reported assembly contiguity (549 contigs with a contig N50 of 106.6 Mb) is impressive, particularly for a large (~3.6 Gb) plant genome assembled using Oxford Nanopore sequencing data. However, several aspects of the assembly strategy and downstream analyses require further clarification or discussion before the manuscript can be considered for publication. 1. Assembly strategy and polishing The genome assembly was generated exclusively from Oxford Nanopore reads without incorporating short-read polishing. While recent ONT chemistry has substantially improved raw read accuracy, the absence of additional polishing may still affect consensus accuracy, particularly in coding regions. The authors are encouraged to either incorporate a modest amount of Illumina sequencing data for polishing or apply an additional long-read polishing step (e.g., Medaka) if feasible. If no further polishing is performed, this limitation should be explicitly discussed, together with its potential implications for the accuracy of the predicted 47,913 protein-coding genes. 2. Genome size estimation The manuscript reports an estimated genome size of approximately 3.2 Gb, whereas the final assembly spans 3.61 Gb. The authors should clarify how the genome size estimate was obtained. If a k-mer-based approach was used, the corresponding k-mer distribution and estimation results should be provided. Alternatively, if the discrepancy reflects retained heterozygous regions or incomplete haplotig removal, this should be discussed, particularly with respect to the haplotig purging procedure employed during assembly. 3. Gene annotation The structural annotation relied primarily on homology evidence from Arabidopsis thaliana together with protein sequences from Heliantheae species. Given the considerable evolutionary distance between Arabidopsis and Asteraceae, as well as the prevalence of whole-genome duplication within this family, the absence of species-specific transcriptomic evidence (RNA-seq or Iso-Seq) may reduce annotation accuracy, particularly for gene structure prediction and splice site identification. Although the authors implemented filtering steps to remove transposable element-related and short gene models, a brief discussion of this limitation and its potential impact on the annotation would strengthen the manuscript. 4. Comparative genomic analysis The comparative genomic analysis is currently limited to reporting an overall alignment rate (82.2%) with S. atractyloides. Including a whole-genome synteny analysis would substantially enhance this section. Such an analysis would provide an independent assessment of assembly continuity and structural accuracy while offering additional insights into chromosome-scale conservation and genome evolution within the genus Scalesia.RecommendationMinor Revision


# Reviewer report: Review MS


Reviewer name and names of any other individual's who aided in reviewer Xiaoxia ZhangDo you understand and agree to our policy of having open and named reviews, and having your review included with the published papers. (If no, please inform the editor that you cannot review this manuscript.)YesIs the language of sufficient quality?YesPlease add additional comments on language quality to clarify if needed
Are all data available and do they match the descriptions in the paper? YesAdditional CommentsAre the data and metadata consistent with relevant minimum information or reporting standards? See GigaDB checklists for examples <a href="http://gigadb.org/site/guide" target="_blank">http://gigadb.org/site/guide</a>NoAdditional CommentsThe authors have deposited genome‑assembly datasets in the NCBI database, whereas relevant data were not submitted to the GigaDB. In addition, we recommend the authors provide gene‑annotation files in standard GFF3 format rather than the GBFF format. GFF3 is the prevailing, community‑accepted annotation format for downstream bioinformatic analysis, and it possesses superior compatibility with most mainstream genome‑analysis pipelines and bioinformatics software. Please supplement the GFF3‑formatted annotation file and complete genome‑data submission to GigaDB before revision.Is the data acquisition clear, complete and methodologically sound?YesAdditional CommentsIs there sufficient detail in the methods and data-processing steps to allow reproduction?YesAdditional CommentsIs there sufficient data validation and statistical analyses of data quality? NoAdditional CommentsTo better illustrate the quality of the present genome assembly, we recommend supplementing a comparative statistics table containing assembly indicators from other closely‑related species.Is the validation suitable for this type of data?YesAdditional CommentsIs there sufficient information for others to reuse this dataset or integrate it with other data?YesAdditional CommentsAny Additional Overall Comments to the AuthorThe authors have reported genomic data for an endemic-endangered plant species in Scalesia genus , which is of great significance for the subsequent conservation work of S. gordilloi. Nevertheless, it is a pity that the genome assembly has not reached the chromosome‑level. To improve the usability of the dataset, the full‑length functional‑gene annotation file is required.RecommendationMinor Revision


# Editor report: Accept


Editor’s AssessmentThe manuscript is ready for formal acceptance.Editor’s AssessmentThe manuscript is ready for formal acceptance.
